# Type II diabetes patients in primary care: profiles of healthcare utilization obtained from observational data

**DOI:** 10.1186/1472-6963-13-7

**Published:** 2013-01-04

**Authors:** Christel E van Dijk, Trynke Hoekstra, Robert A Verheij, Jos WR Twisk, Peter P Groenewegen, François G Schellevis, Dinny H de Bakker

**Affiliations:** 1 NIVEL, Netherlands Institute for Health Services Research, P.O.Box 1568, Utrecht, 3500 BN, The Netherlands; 2 VU University, Faculty of Earth- and Life Sciences, Department of Health Sciences and the EMGO Institute for Health and Care Research, Boelelaan 1085, Amsterdam, 1081 HV, The Netherlands; 3 VU University medical center, Department of Epidemiology and Biostatistics and the EMGO Institute for Health and Care Research, Boelelaan 1085, Amsterdam, 1081 HV, The Netherlands; 4 Utrecht University, Department of Sociology, Department of Human Geography, P.O.Box 80140, Utrecht, 3508 TC, The Netherlands; 5 VU University medical center, Department of General Practice and the EMGO Institute for Health and Care Research, Van der Boechorstraat 7, Amsterdam, 1081 BT, The Netherlands; 6 Tilburg University, Scientific Centre for Transformation in Care and Welfare (TRANZO), Tilburg 90153, 5037 AB, The Netherlands

**Keywords:** Type II diabetes mellitus, Healthcare utilisation profiles, Primary care, Latent Class Analyses

## Abstract

**Background:**

The high burden of diabetes for healthcare costs and their impact on quality of life and management of the disease have triggered the design and introduction of disease management programmes (DMPs) in many countries. The extent to which diabetes patients vary with regard to their healthcare utilisation and costs is largely unknown and could impact on the design of DMPs. The objectives of this study are to develop profiles based on both the diabetes-related healthcare utilisation and total healthcare utilisation in primary care, to investigate which patient and disease characteristics determine ‘membership’ of each profile, and to investigate the association between these profiles.

**Methods:**

Data were used from electronic medical records of 6721 known type II diabetes patients listed in 48 Dutch general practices. Latent Class Analyses were conducted to identify profiles of healthcare and regression analyses were used to analyse the characteristics of the profiles.

**Results:**

For both diabetes-related healthcare utilisation and total healthcare utilisation three profiles could be distinguished: for the diabetes-related healthcare utilisation these were characterised as ‘*high utilisation and frequent home visits*’ (n=393), ‘*low utilisation, GP only*’ (n=3231) and ‘*high utilisation, GP and nurse*’ (n=3097). Profiles differed with respect to the patients’ age and type of medication; the oldest patients using insulin were dominant in the ‘high utilisation, GP and nurse’ profile. High total healthcare utilisation was not associated with high diabetes-related healthcare utilisation.

**Conclusions:**

Healthcare utilisation of diabetes patients is heterogeneous. This challenges the development of distinguishable DMPs.

## Background

The number of people with type II diabetes mellitus is increasing [1]. Due to the high burden of diabetes in particular and chronic diseases in general for healthcare costs and their impact on quality of life, management of these diseases has become an important issue in health policy in many countries [2]. This has triggered the design and introduction of disease management programmes (DMPs) for type II diabetes mellitus in particular. According to the Disease Management Association of America (DMAA) disease management is defined as a system of coordinated healthcare interventions and communications for populations with conditions in which patients’ self-care efforts are significant. Disease management supports the physician or practitioner/patient relationship and plan of care, emphasizes prevention of exacerbations and complications through the use of evidence-based practice guidelines and patient empowerment, and evaluates clinical, humanistic, and economic outcomes on an ongoing basis with the goal of improving overall health [3]. DMPs are expected to be a solution for the inadequate coordination of care between health services, variation in quality of care and increasing costs for chronic illnesses [4]. However, literature on the design and effects on health and disease outcomes is inconclusive and research currently focuses mainly on refining these issues, such as defining the optimal patient group per programme [5,6].

Remuneration of DMPs differs between European countries: a yearly price for total care for a chronic disease (e.g. Denmark, UK, the Netherlands), a financial bonus for general practitioners (GPs) per patient that is included in a DMP (e.g. France), dedication of one percent of the total health care budget and refunding additional services for DMP-patients (e.g. Germany) [7]. A position paper showed that providing financial incentives to relevant stakeholders is important for facilitating successful implementation of DMPs [4]. Stakeholders may be reluctant to invest in better chronic care if their investments are not accompanied by better payment, or at least equal compensation [8]. Setting the incentives correctly will encourage healthcare providers to efficiently provide healthcare services. In the case of DMPs, varying healthcare demands between and within patients over time might make it problematic to design a good financial compensation system for DMPs. If physicians are paid an equal amount per patient, they might be reluctant to include patients with a high healthcare demand and consequently risk of financial losses or risk of under-treatment [9]. On the other hand, a fee-for-service remuneration system might provide the incentive for physicians to increase the number of services, with a risk for increased costs and over-treatment [9,10]. Incorporating possible heterogeneity of patients’ healthcare demands in the design of DMPs and remuneration of professionals might be a solution to this problem. However, to what extent diabetes patients vary with regard to their healthcare utilisation and costs is largely unknown. El Fakiri et al. distinguished healthcare utilisation patterns for diabetes patients in the Netherlands [11]. However, these patterns were based on the total healthcare utilisation of diabetes patients and did not distinguish between type I and type II diabetes patients, although it is known that healthcare utilisation of type I and type II diabetes patients is different [11-13]. Moreover, DMPs have been focusing mainly on just one disease and not on multiple diseases. Most diabetes patients, however, also suffer from other chronic diseases. Sixty percent of diabetes type II patients have at least one other chronic disease and twelve percent of the patients even have three or more other chronic conditions [14]. Although it might be more realistic to incorporate all utilised healthcare instead of diabetes-related healthcare utilisation only in DMPs, we do not know whether diabetes patients with a high healthcare demand related to diabetes also have a higher demand for healthcare in general and, if so, whether this is equally true for all diabetes patients.

The objective of this study was therefore to empirically develop profiles of healthcare use of type II diabetes patients based on both the total healthcare utilisation and diabetes-related healthcare utilisation in primary care; to determine which patient and disease characteristics determine the ‘membership’ of each profile; and to assess the association between profiles of total healthcare utilisation and diabetes-related utilisation. Such empirically derived profiles may be useful when designing DMPs and planning remuneration of professionals.

## Methods

### Study design

For the purpose of this retrospective study, primary care utilisation of known diabetes patients was assessed in 2008 by developing profiles of diabetes-related healthcare utilisation and of total health-care utilisation separately. Data from the Netherlands Information Network of General Practice (LINH) were used, which is a representative sample of GP-practices in the Netherlands that provide routinely recorded data from their electronic medical records (EMRs) of all patients listed in their practice. The Dutch healthcare system is very useful for analysing longitudinal data. All Dutch inhabitants are obligatory listed in a general practice and the GP acts as gatekeeper for specialized health care. Therefore, the EMR kept by the GP is the most complete record. The LINH-database holds longitudinal data on morbidity, drug prescriptions and referrals of approximately 90 GP-practices and 350,000 listed patients [15]. The network is a dynamic pool of practices, with each year some minor changes in the composition of practices. Diagnoses are coded by the GPs using the International Classification of Primary Care (ICPC) [16]. LINH is registered with the Dutch Data Protection Authority; data are handled according to the data protection guidelines of the authority. According to the Dutch legislation, ethical approval is not required for observational studies.

For our analyses, we used data from practices that a) participated in both 2007 (for determining known diabetes patients) and 2008 and b) provided recorded year-round data for consultations, prescriptions, and morbidity and referral records in 2008.

Patients were selected for this study if 1) they had consulted their GP for type II diabetes at least once in 2007 and 2) were registered with the practice during the whole year in 2008 and 3) were 18 or older. Type II diabetes patients were selected on the basis of a recorded ICPC-code T90. GPs participating in LINH do generally not record on ICPC sub codes (T90.1 or T90.2), and therefore we could not distinguish between type I and type II diabetes patients on the basis of ICPC-codes. For the purpose of this study, type I diabetes patients were excluded on the basis of having received a prescription of insulin (ATC-code A10A), but not any oral anti-diabetic medication (ATC-code A10B) [14,17]. In total, data of 48 GP-practices and 6,721 type II diabetes patients were included. Reasons for exclusion were 1) incomplete data on consultations (16% of practices), 2) incomplete data on prescriptions (28%) and/or 3) incomplete data on referrals (44%).Overall, these GP-practices were representative of the Dutch GP-practices with respect to degree of urbanisation and region, but not with respect to practice type (overrepresentation of group practices or health centres, underrepresentation of single handed practices).

### Healthcare utilisation in primary care

Healthcare utilisation of subjects consisted of contacts with general practice, drug prescriptions and referrals to allied healthcare: primary healthcare and medication. Healthcare utilisation was regarded as diabetes-related, if the care provided was mentioned in the multidisciplinary healthcare standard of the Dutch Diabetes Federation (NDF: “Nederlandse Diabetes Federatie”) [18]. We only took into account healthcare utilisation for which an ICPC code was recorded by the GP. Additional file 1 shows the definition of diabetes related healthcare for known type II diabetes patients with the corresponding ICPC-codes.

Contacts in general practice were derived from claims data in the EMR in which a distinction was made between consultations in the practice, home visits and telephone consultations with GPs and primary care nurses. The numbers of consultations, home visits and telephone consultations with both GPs and primary care nurses separately, were included in the analyses.

Drug prescriptions were coded using the ATC classification system. Prescriptions mentioned in de NDF-guideline (see Additional file 1) as well as prescriptions related to the diabetes-related health problems were included in the analyses of diabetes-related profiles. A list of included drug prescriptions is provided in Additional file 2. The number of different drug prescriptions based on ATC codes at the 4 digit level was included in the analyses. If a prescription could be captured with an ATC code at the 5 digit level, the ATC code at the 5 digit level was included.

Healthcare utilisation of patients with allied healthcare providers was estimated on the basis of GP referrals. Referrals for diabetes-related health problems to a physiotherapist or exercise therapist, dietician and podiatrist were included in the analyses.

### Patient and disease characteristics

Patient and disease characteristics included in this study were age (categorised), gender, urbanisation (categorised), diabetes medication type (no treatment, oral treatment or oral treatment and insulin) and comorbidity; all data were derived from the EMR. Comorbidity was divided into several categories of related and unrelated comorbidity according to Struijs et al. (2006) [19]. The following comorbid diseases were regarded as diabetes-related: (with ICPC-codes): heart diseases (K74-K76), stroke (K90), retinopathy (F83), nephropathy (U99) and diabetic foot (K99.06 and N94; deviated from Struijs et al.). Non-related comorbidity included depression (P76), lung diseases (R91, R95, and R96), musculoskeletal diseases (L01-L03, L08, L13, L15, L84, L86, and L89-91), neurological diseases (N86-N88) and cancer (B74, D74, D75, D77, R84, S77, X76, Y77).

### Statistical analyses

To analyse the different profiles, Latent Class Analyses (LCA) were performed to identify distinct classes of patients with specific combinations of healthcare utilisation. LCA is a type of cluster analysis used to group patients into *k* number of unique (otherwise unobserved) categories, where, within each category patients are most similar to each other regarding their healthcare utilisation, and between the categories patients are most different [20-22]. To find the optimal number of categories, a 2–6 class solution was modelled and output was assessed and compared according to a stepwise approach described elsewhere [20,22]. To determine the final solution several model fit indicators were used [23]. The Bayesian Information Criterion (BIC) (where a lower BIC indicates a better fit) and posterior probabilities (where probabilities close to 1 indicates a better classification and posterior probabilities at least 0.8 are advised [24,25]) were used as model fit indicators. Also, we assessed the usefulness and clinical interpretation of each solution. The usefulness was assessed by considering the solutions based on the number of people in each class (hereby rejecting solutions with small groups: minimum N = 200). Mplus was used to perform LCA because within Mplus, LCA can adequately cluster a combination of both categorical (also binary variables) and count data [26]. LCA was conducted for both diabetes-related healthcare utilisation and total healthcare utilisation separately. Each profile was given a label resembling their healthcare utilization. Subsequently, a predictive model was made using multilevel multinomial regression analyses (patients nested in practices) for the diabetes-related healthcare utilisation profiles. In this analysis, it was assessed whether patient and disease characteristics were associated to profile membership. Analyses were performed using STATA, Mplus and MLwiN.

## Results

For both the diabetes-related primary healthcare and total primary healthcare, a three-class solution was found to be the best model according to the criteria described in the statistical analyses section above (BIC: 102255, posterior probabilities 0.969, 0.941 and 0.963 respectively and BIC: 148819 and posterior probabilities 0.913, 0.949 and 0.917 respectively). Table 1 presents the descriptive information of the profiles regarding healthcare utilisation. Table 2 describes the diabetes-related healthcare-profiles in terms of patient and disease characteristics.

**Table 1:** Description of profiles of diabetes-related and total primary healthcare utilisation of diabetes type II patients

	**Diabetes-related primary healthcare**				**Total primary healthcare**		
	**Profile 1**	**Profile 2**	**Profile 3**	**Profile 1**	**Profile 2**	**Profile 3**
	**‘**high utilisation and frequent home visits’**(n=393)**	‘low utilisation, GP only’**(n=3231)**	‘high utilisation, GP and nurse’ **(n=3097)**	‘low utilisation, GP only’**(n =2669)**	‘medium-high utilisation, GP and nurse”**(n=2929)**	‘high utilisation’ **(n=1123)**
	*mean (95% CI)*	*mean (95% CI)*	*mean (95% CI)*	*mean (95% CI)*	*mean (95% CI)*	*mean (95% CI)*
*Number per year*						
GP contacts	0.45 (0.34-0.59)	1.65 (1.30-2.10)	1.59 (1.27-1.98)	4.38 (3.84-4.99)	4.34 (3.65-5.17)	5.67 (4.37-7.36)
GP home visits	1.08 (0.88-1.32)	0.19 (0.14-0.27)	0.05 (0.02-0.10)	0.15 (0.09-0.26)	0.09 (0.06-0.14)	3.95 (3.07-5.07)
GP telephone consultation	0.95 (0.70-1.30)	0.28 (0.21-0.38)	0.30 (0.24-0.39)	0.81 (0.58-1.11)	0.86 (0.69-1.08)	4.15 (3.55-4.85)
Primary care nurse contacts	0.41 (0.27-0.63)	0.09 (0.04-0.21)	3.48 (3.01-4.03)	0.08 (0.04-0.20)	3.75 (3.33-4.23)	1.15 (0.58-2.31)
Primary care nurse home visits	2.71 (2.23-3.29)	0.00 (0.00-0.01)	0.69 (0.50-0.94)	0.00 (0.00-0.01)	0.02 (0.01-0.04)	1.27 (0.83-1.94)
Primary care nurse telephone consultation	1.99 (1.13-3.50)	0.01 (0.00-0.03)	0.68 (0.47-0.98)	0.01 (0.00-0.03)	0.60 (0.40-0.91)	1.26 (0.72-2.23)
Number of different prescription (ATC4-level)	4.42 (4.14-4.69)	3.28 (3.13-3.43)	3.68 (3.52-3.84)	6.70 (6.12-7.28)	7.23 (6.62-7.84)	13.28 (12.65-13.91)
*Referral to (%)*						
Dietician	1.6%	1.0%	1.3%	1.6%	2.8%	2.5%
Physiotherapist	4.3%	3.3%	4.9%	5.7%	8.6%	13.7%
Podiatrist	1.5%	0.3%	1.4%	4.2%	2.9%	2.6%

**Table 2:** Patient and disease characteristics of profiles of diabetes-related primary healthcare of diabetes type II patients

	**Diabetes-related primary healthcare**		
	**Profile 1**	**Profile 2**	**Profile 3**
	**‘**high utilisation and	‘low utilisation, GP only’	‘high utilisation,
	frequent home	**(n=3231)**	GP and nurse’
	visits’		**(n=3097)**
	**(n=393)**		
Gender (% female)	73.5	50.8	48.0
*Age*			
18-34	0.3	1.7	0.5
35-44	0.8	5.9	3.7
45-54	1.0	14.3	13.2
55-64	3.3	26.3	29.4
65-74	16.8	26.1	30.6
75 and older	77.9	25.7	22.5
Urbanisation			
2500 or more addresses per square km	27.0	26.5	13.2
1500-2499 addresses per square km	20.9	27.3	31.8
1000-1499 addresses per square km	20.9	21.8	13.3
500-999 addresses per square km	14.5	11.3	15.7
<500 addresses per square km	16.8	13.1	26.0
*Main medication*			
No medication	16.0	32.3	25.1
Oral medication only	61.3	54.6	61.6
Oral medication and insulin	22.7	13.1	13.3
*Related comorbidity*			
Heart diseases	21.4	10.5	15.2
Stroke	10.7	3.3	3.2
Retinopathy	1.0	0.5	0.6
Nephropathy	6.6	1.9	2.6
Diabetic foot	2.8	2.1	1.8
*Non-related comorbidity*			
Depression	8.7	3.9	4.3
Lung diseases	18.1	9.0	12.0
Musculoskeletal diseases	36.4	27.0	28.0
Neurological diseases	2.0	1.2	1.1
Cancer	8.1	3.7	3.4

### Profiles of diabetes-related primary healthcare utilisation

In the first three columns of Table 1 the descriptive information of the profiles regarding diabetes-related healthcare is presented. The first profile ‘high utilisation and frequent home visits’ was mainly characterised by a high number of home visits and telephone consultations by both GPs and primary care nurses. Consultations in the practice were less common for diabetes patients in this profile. The second profile ‘low utilisation, GP only’ was characterised by a relatively low number of consultations. Patients in profile 3 ‘high utilisation, GP and nurse’ were characterised by a high number of consultations in the practice, especially frequent consultations with primary care nurses. Diabetes patients in profile ‘high utilisation and frequent home visits’ had on average 4.7 face-to-face contacts related to diabetes with GPs and/or primary care nurses, patients in profile ‘low utilisation, GP only’ 1.9 and diabetes patients in profile ‘high utilisation, GP and nurse’ had 5.8 contacts.

Table 2 shows the comparison of the three profiles with regard to patient and disease characteristics; Table 3 shows the results of the multinomial logistic regression analysis. Clearly, diabetes patients in profile 1 were significantly older, mostly female, more of them had had a stroke, and had a significantly higher prescription rate of both oral medication only and of the combination of oral and insulin medication. Patients in profile 2 and profile 3 were difficult to distinguish; the only clear difference was the age range and medication usage, with the youngest patients and patients with no diabetes medication classified in profile 2.

**Table 3:** Results of multilevel multinomial logistic regression analysis predicting cluster membership

	**Diabetes-related healthcare**		
	**Profile 1** ‘high utilisation and frequent home visits’	**Profile 3** ‘high utilisation, GP and nurse’	**Profile 1** ‘high utilisation and frequent home visits’
	**(Profile 2** ‘low utilisation, GP only’**= reference group)**	**(Profile 2** ‘low utilisation, GP only’**= reference group)**	**(Profile 3** ‘high utilisation, GP and nurse’**= reference group)**
**Patient and disease characteristics**	**Odds ratio (95% CI)**	**Odds ratio (95% CI)**	**Odds ratio (95% CI)**
Gender (reference male)	**1.80 (1.38-2.34)**	0.92 (0.83-1.02)	**2.27 (1.76-2.93)**
*Age (reference 18–44)*			
45-54	0.43 (0.09-1.92)	**1.46 (1.12-1.90)**	0.35 (0.07-1.72)
55-64	0.71 (0.21-2.41)	**1.64 (1.29-2.10)**	0.48 (0.13-1.82)
65-74	**3.24 (1.08-9.69)**	**1.74 (1.36-2.22)**	2.22 (0.67-7.39)
75 and older	**16.20 (5.55-47.27)**	1.26 (0.98-1.62)	**15.47 (4.76-50.27)**
Urbanisation (reference: 2500 or more addresses per square km)			
1500-2499 addresses per square km	**0.61 (0.38-0.99)**	1.19 (0.94-1.52)	**0.57 (0.36-0.90)**
1000-1499 addresses per square km	0.69 (0.40-1.21)	1.11 (0.83-1.48)	0.65 (0.38-1.10)
500-999 addresses per square km	0.58 (0.30-1.12)	1.14 (0.82-1.60)	**0.53 (0.28-1.00)**
<500 addresses per square km	0.66 (0.34-1.29)	1.17 (0.80-1.72)	0.60 (0.32-1.15)
*Main medication form (reference: no medication)*			
Oral medication only	**2.16 (1.58-2.96)**	**1.61 (1.43-1.82)**	**1.53 (1.13-2.06)**
Oral medication and insulin	**3.46 (2.35-5.10)**	1.15 (0.96-1.37)	**3.24 (2.25-4.67)**
*Related comorbidity*			
Heart diseases	1.04 (0.76-1.44)	0.99 (0.85-1.15)	1.14 (0.85-1.53)
Stroke	**1.90 (1.25-2.89)**	0.76 (0.57-1.01)	**2.92 (2.00-4.27)**
Retinopathy	1.28 (0.37-4.50)	1.43 (0.73-2.82)	0.55 (0.14-2.18)
Nephropathy	1.34 (0.78-2.28)	0.88 (0.62-1.24)	**1.65 (1.00-2.70)**
Diabetic foot	1.01 (0.48-2.11)	0.88 (0.61-1.27)	1.21 (0.62-2.36)
*Non-related comorbidity*			
Depression	1.50 (0.96-2.35)	1.03 (0.80-1.32)	**1.72 (1.15-2.56)**
Lung diseases	**1.44 (1.04-1.98)**	1.00 (0.85-1.18)	**1.40 (1.03-1.89)**
Musculoskeletal diseases	1.20 (0.94-1.55)	1.05 (0.94-1.17)	1.20 (0.95-1.51)
Neurological diseases	1.06 (0.45-2.49)	0.75 (0.46-1.22)	1.89 (0.91-3.91)
Cancer	1.34 (0.84-2.14)	0.80 (0.61-1.06)	**1.84 (1.20-2.82)**

Although corrected for patient and disease characteristics, large practice variance still existed with regards to profile membership. For example, for 13 practices all patients were assigned to profile 2, and for one practice all patients were assigned to profile 3. General practices with patients assigned in profile 2 had less often a primary care nurse working in the practice, were more often single handed practices and less often group practices compared to general practices with more variation in patients’ profiles.

### Profiles of total primary healthcare utilisation

The last three columns of Table 1 show the descriptive information of the profiles including total primary healthcare. The first profile ‘low utilisation, GP only’ was characterised by GP-consultations in the practice only in combination with a relatively low number of different drug prescriptions and referrals, although 4.2% of the patients were referred to a podiatrist. The second profile ‘medium-high utilisation, GP and nurse’ was also characterised by a high number of GP-consultations in the practice, and by a higher number of consultations with a primary care nurse in the practice. Patients in profile 3 ‘high utilisation’ were particularly characterised by contacts with a GP in practice and home visits by both GP and nurse. Moreover, patients in this profile were characterised by a high prescription rate and referrals to physiotherapists. Diabetes patients in profile ‘low utilisation, GP only’ had on average 4.6 face-to-face contacts with GPs and/or primary care nurses, patients in profile ‘consultation by GP and nurse’ 8.2 and diabetes patient in the profile ‘high utilisation’ 12.0 contacts.

### Comparing membership of profiles of diabetes-related primary care with membership of profiles of total primary healthcare utilisation

Table 4 shows the cross tabulation between the diabetes-related primary care profiles and total primary healthcare profiles. Low healthcare utilisation for total primary healthcare (profile 1) was associated with a low diabetes-related healthcare utilisation (profile 2), whereas a high total healthcare utilisation (profile 3) did not necessarily imply a high healthcare utilisation profile for diabetes-related primary healthcare. Comparing the two profiles with high diabetes-related primary healthcare utilisation (profile 1 and 3) for diabetes patients with the total primary healthcare profile ‘high utilisation’ showed that diabetes patients in the ‘high utilisation and frequent home visits’ profile (n=386) were more often women and aged 75 year or older, and diabetes patients in the ‘high utilisation, GP and nurse’ (n=297) more often had diabetes related comorbidity (heart disease and stroke) and unrelated comorbidity (lung- and musculoskeletal diseases).

**Table 4:** Cross tabulation of profiles of diabetes-related and total primary healthcare utilisation of diabetes type II patients

	**Diabetes-related primary healthcare**		
**Total primary healthcare**	**Profile 1** ‘high utilisation and frequent home visits’ (n=393)	**Profile 2** ‘low utilisation, GP only’ (n=3231)	**Profile 3** ‘high utilisation, GP and nurse’ (n=3097)
**Profile 1**	4	2661	4
‘low utilisation, GP only’ (n=2669)			
**Profile 2**	3	130	2796
‘ medium-high utilisation, GP and nurse” (n=2929)			
**Profile 3**	386	440	297
‘high utilisation’ (n= 1123)			

## Discussion

The purpose of this study was to develop profiles based on both total and diabetes-related primary healthcare utilisation and to investigate the association between profiles of total healthcare utilisation and diabetes-related utilisation. For both diabetes and total primary healthcare utilisation, three clearly distinct profiles were found with regard to the type of contacts and type of healthcare provider (GP or primary care nurse). Patient and disease characteristics were, however, not always associated with the membership of each profile. Age and type of medication – no medication, oral medication or oral medication and insulin – were the strongest indicators for diabetes-related primary healthcare profiles. Diabetes patients with a high total healthcare utilisation (profile ‘high utilisation’), were not always patients with a high utilisation pattern for diabetes (‘high utilisation, GP and nurse’), whereas having a low total healthcare utilisation profiles was associated with a low contact rate for diabetes.

### Profiles of diabetes-related primary care utilisation

According to the guidelines, type II diabetes patients under supervision of GPs should have four regular check-ups within the practice per year [18]. Of our three diabetes-related primary healthcare utilisation profiles, only the patients in profile ‘low utilisation, GP only’ had on average less than the recommended four contacts for diabetes-related issues. Interestingly, this profile represented almost half of the type II diabetes population in general practice. Principal treatment in secondary care (by an internist – in the Netherlands internists are not seen as primary care specialists) could explain the low number of contacts in primary care for part of this subgroup, but we do not have information available in our dataset to confirm this. However, from a report published by the National Institute for Public Health and the Environment (RIVM), it is known that only a small number of type II diabetes patients is under treatment solely by an internist [27], thereby possibly not providing a full explanation for our findings. Thus, part of the type II diabetes patients did not have the recommended four contacts annually for diabetes-related health problems. In general, the patients in the profile with low frequency of contacts are the youngest in the sample, and also show the lowest prevalence of co-morbidity. This might coincide with well-controlled diabetes, indicating a less-frequent need for primary care consultations [28]. In this respect, our findings showing that patients from a quarter of the practices were all assigned to the 'low utilisation, GP only' profile, are notable. It might be that these practices do not provide adequate care to type II diabetes patients, which may be explained by unavailability of primary care nurses.

We showed that diabetes-related primary healthcare utilisation is heterogeneous. Only one previous study has researched this heterogeneous presentation also [11]. This study, conducted in a much smaller sample of Dutch diabetes patients (around 400 patients), included both type I and type II diabetes patients and total care utilised in both primary and secondary care, found four distinct profiles of healthcare utilisation. Although difficult to compare due to methodological issues, our profiles point to a fairly similar picture; for example, we also find a large subgroup of patients with low healthcare utilisation.

### Determinants of diabetes-related primary healthcare profile membership

Diabetes-related primary healthcare profiles could only partly be explained by patient and disease characteristics. Age and use of oral medication and insulin were the strongest predictors for membership in a diabetes-related primary healthcare profile with high utilisation. In agreement with our study, El Fakiri et al. also found, except for the type of diabetes, little effect of possible predictors for the different healthcare profiles. However, this study investigated other predictors than we did [11]. They did show that the patients classified into the profile with the highest number of contacts in general practice more often had comorbidity, which was not consistently found in our study. However, this difference can be explained by the fact that our membership of the profiles was determined on diabetes-related primary healthcare utilisation only. These results illustrate the difficulty of predicting healthcare utilisation for diabetes patients. With the consequence that it is also problematic to develop different DMP for diabetes type II based on patient and disease characteristics, since it does not resemble the healthcare utilisation and therefore costs. In conclusion, these results do not assist health planners in allocating diabetes type II patients in different DMPs. However, differentiations in the remuneration system for patients with differing healthcare demands might also lead to an unnecessary complexity in the design of such DMPs, coinciding with an increase in administrative costs.

### Association between diabetes-related and total primary healthcare utilisation

Our study showed that a high total primary healthcare utilisation profile was not generally associated with a high diabetes-related primary healthcare utilisation profile. Both age and the existence of related and unrelated comorbidity were determinants for having both a higher total and diabetes-related primary healthcare utilisation profile. This is in accordance with previous research that showed that both diabetes-related and diabetes-unrelated comorbidity increased the use of medical care in diabetes patients [19]. A recent review also showed a positive association between multiple chronic conditions and healthcare utilisation and expenditure [29]. These results indicate that total primary healthcare utilisation is not a good indicator for disease specific healthcare utilisation. When incorporating total utilised healthcare instead of diabetes-related healthcare utilisation only in a DMP, specific attention should be paid to the role of age and comorbidity, in particular as only these patient characteristics predict high healthcare utilisation for both total and diabetes-related care.

### Strengths and limitations

The empirical derivation of profiles of healthcare utilisation of type II diabetes patients as opposed to self-defined profiles provides new insights in healthcare utilisation and demands of type II diabetes patients. A number of points should be considered in our study. First, not all GPs’ actions were recorded in a structured way in their EMR and could for that reason not be incorporated in our analyses. We chose to include only the information that was recorded in a concise and structured way by all GPs. This meant that we unfortunately were unable to include information about the exact content of the consultations in general practice and do not know whether for example lifestyle advice was given, nor were structured clinical outcome data available (e.g. glycated haemoglobin level or blood pressure). This then makes it impossible to make inferences about the effect of the different primary healthcare utilisation profiles on patient outcomes. This should be addressed in future research. Second, no referral to a physiotherapist is needed since 2006 and therefore the number of patients visiting a physiotherapist was underestimated. Research shows that mostly patients with acute problems (instead of chronic problems) visit a physical therapist on their own initiative, which is not often the case with diabetes patients [30]. In some practices no primary care nurse was working in the practice, and therefore these patients may not be assigned to profiles which are largely described by contacts with primary care nurses. Additional analyses (available upon request by the first authors) limited to practices with a primary care nurse showed similar effects of determinants of diabetes-related primary healthcare profile membership, although the profile ‘high utilisation, GP and nurse’ in comparison to the profile ‘low utilisation, GP only’ showed slightly underestimated effects of the main medication type for diabetes compared to the model with all practices. In addition, healthcare utilisation as presented in this study does not reflect the ideal or needed level of healthcare.

### Implications of the findings

DMPs are expected to be the solution for the inadequate coordination of care, variation in quality of care and increasing costs for illnesses [4]. The design of such standardised programmes ultimately requires a homogeneous patient population or in case of a heterogeneous population at least one that is easily explained by clear patient and disease characteristics. Unexplained heterogeneity in healthcare demands of these patients, therefore, means that a standardised programme might be insufficient or inadequate for some patients. Moreover, a heterogeneous patient population with diverse healthcare demands might cause physicians to be reluctant in the inclusion of patients with high healthcare demands. The issue of multimorbidity is also predominant in type II diabetes patients. This results in the fact that a large part of the healthcare utilisation of these patients might not be included in diabetes DMPs if these DMPs would focus exclusively on diabetes neglecting other existing health problems [14]. With a non-explained heterogeneous diabetes type II population and large non-diabetes-related primary healthcare utilisation, health planners might consider putting more emphasis on case management instead of disease management.

## Conclusions

In conclusion, we have shown that primary healthcare utilisation of diabetes patients is heterogeneous, which could be captured in three distinct profiles of diabetes-related and total healthcare utilisation. The diabetes-related profiles were only partly explained by patient and disease characteristics, posing difficulties for the future development of distinguishable disease management programmes. Further, we have shown that total primary healthcare utilisation is not a good indicator for diabetes-related primary healthcare utilisation for diabetes patients. This fact should also be taken into account in the remuneration system of DMPs.

## Competing interests

The authors declare that they have no competing interests.

## Authors’ contributions

CD and TH were involved in the conception of the research question and in analysing the data. All authors had full access to all the data and contributed to the interpretation of the data. CD and TH drafted the manuscript, which was reviewed by all authors. All authors read and approved the final manuscript.

## Pre-publication history

The pre-publication history for this paper can be accessed here:

http://www.biomedcentral.com/1472-6963/13/7/prepub

## Supplementary Material

Additional file 1 Healthcare utilisation for known type II diabetes patients based on Dutch Diabetes Federation type II diabetes guideline. Click here for file

Additional file 2 Prescriptions related to diabetes care. Click here for file
