# Supplementary material for: Type II diabetes patients in primary care: profiles of healthcare utilization obtained from observational data
Source: BMC Health Serv Res. 2013 Jan 4;13:7. doi: 10.1186/1472-6963-13-7 (PMC3570342; doi:10.1186/1472-6963-13-7)
Supplement: Additional file 1 — Healthcare utilisation for known type II diabetes patients based on Dutch Diabetes Federation type II diabetes guideline. [file 1472-6963-13-7-S1.doc]

**ATTACHMENT I: Healthcare utilisation for known type II diabetes patients based on Dutch Diabetes Federation type II diabetes guideline**

*Check-ups by GP and primary care nurse*

3-montly check-up: wellbeing, hypo- or hyperglycemia, nutritional problems or exercise advice and medication, body weight, fasting blood glucose levels, blood pressure (if patient uses antihypertensive drugs), foot examination (if patient had ulcus, acquired deformity of limb or serious neuropathy foot)

Yearly check-up : possible visual problems , cardiovascular diseases, neuropathy or sexual problems, lifestyle aspects as smoking status, exercise and alcohol use, blood pressure, body weight, foot examination, inspection insulin injection sites (if patients uses insulin), eye fundus examination, Laboratory measures: fasting blood glucose, HbA1c, creatinine levels, potassium levels (if patient uses diuretic or RAS inhibitor), creatinine clearance, albumin creatinine-ratio or albumin urine levels (if patients has a life expectancy of minimal 10 years), fasting lipids spectrum.

*Medication*

Diabetes: oral blood glucose lowering drugs, insulin

Risk factor cardiovascular diseases: lipid modifying agents (recommended for almost all type II diabetes patients), diuretics, ACE inhibitors, angiotensin-ii-antagonists, Beta blocking agents, Calcium Channel blockers, Antithrombotic agents

Superficial foot ulcer: oral antibiotic

*Consultation other healthcare providers*

Internist (including nephrologist): adjustment insulin (when knowledge not available in GP-practice), insufficient correction postprandial blood glucose levels with two-times daily insulin, diabetes ulcer, low creatinine clearance, serious hyperglycaemia or hyperglycaemic coma, pregnant women or women with pregnancy wish.

Dietician: for extensive nutrition advice

Ophthalmologist: retina photography (if not available in GP-practice), assessment of retina photography (if expertise not available in GP-practice), deviations eye fundus.

Podotherapist: callous and/or pressure sites without signs of peripheral vascular disease

Surgeon: diabetes ulcer

Orthopaedic: diabetes ulcer

Dermatologist: diabetes ulcer

Based on the described healthcare utilisation the following ICPC-codes were coded as ‘according to the guideline’:

| F05: Visual disturbance other | S06: Rash localized |
| --- | --- |
| F83: Retinopathy | S11: Other local infection skin |
| F94: Blindness | T02: Excessive appetite |
| K74: Angina pectoris | T03: Loss of appetite |
| K75: Acute myocardial infarction | T05: Feeding problem of adult |
| K76: Ischemic heart disease w/o angina | T07: Weight gain |
| K86: Hypertension uncomplicated | T08: Weight loss |
| K87: Hypertension complicated | T82: Obesity |
| K89: Transient cerebral ischemic | T83: Overweight |
| K90: Stroke/ cerebrovascular disease, | T90: Diabetes mellitus |
| K99.06: Peripheral diabetic angiopathy | T93: Lipid disorder |
| L98: Acquired deformity limb | X24: Fear of sexual dysfunction female |
| N94: Peripheral neuritis/neuropathy | Y07: Impotence NOS |
| P07: Sexual desire reduced | Y24: Fear of dysfunction male |
| P08: Sexual fulfilment reduced, |  |
| P17: Tobacco abuse |  |
