# Supplementary material for: Type II diabetes patients in primary care: profiles of healthcare utilization obtained from observational data
Source: BMC Health Serv Res. 2013 Jan 4;13:7. doi: 10.1186/1472-6963-13-7 (PMC3570342; doi:10.1186/1472-6963-13-7)
Supplement: Additional file 2 — Prescriptions related to diabetes care. [file 1472-6963-13-7-S2.doc]

**ATTACHMENT 2: Prescriptions related to diabetes care**

| **ATC-code** | **Description** |
| --- | --- |
| A10A | [Insulins and analogues](http://en.wikipedia.org/wiki/ATC_code_A10#A10A_Insulins_and_analogues%23A10A_Insulins_and_analogues) |
| A10B | [Blood glucose lowering drugs, excluding insulins](http://en.wikipedia.org/wiki/ATC_code_A10#A10B_Blood_glucose_lowering_drugs.2C_excluding_insulins%23A10B_Blood_glucose_lowering_drugs.2C_excluding_insulins) |
| B01A | [Antithrombotic agents](http://en.wikipedia.org/wiki/ATC_code_B01#B01A_Antithrombotic_agents%23B01A_Antithrombotic_agents) |
| C02A | [Antiadrenergic agents, centrally acting](http://en.wikipedia.org/wiki/ATC_code_C02#C02A_Antiadrenergic_agents.2C_centrally_acting%23C02A_Antiadrenergic_agents.2C_centrally_acting) |
| C02B | [Antiadrenergic agents, ganglion-blocking](http://en.wikipedia.org/wiki/ATC_code_C02#C02B_Antiadrenergic_agents.2C_ganglion-blocking%23C02B_Antiadrenergic_agents.2C_ganglion-blocking) |
| C02C | [Antiadrenergic agents, peripherally acting](http://en.wikipedia.org/wiki/ATC_code_C02#C02C_Antiadrenergic_agents.2C_peripherally_acting%23C02C_Antiadrenergic_agents.2C_peripherally_acting) |
| C02D | [Arteriolar smooth muscle, agents acting on](http://en.wikipedia.org/wiki/ATC_code_C02#C02D_Arteriolar_smooth_muscle.2C_agents_acting_on%23C02D_Arteriolar_smooth_muscle.2C_agents_acting_on) |
| C02K | [Other antihypertensives](http://en.wikipedia.org/wiki/ATC_code_C02#C02K_Other_antihypertensives%23C02K_Other_antihypertensives) |
| C02L | [Antihypertensives and diuretics in combination](http://en.wikipedia.org/wiki/ATC_code_C02#C02L_Antihypertensives_and_diuretics_in_combination%23C02L_Antihypertensives_and_diuretics_in_combination) |
| C02N | [Combinations of antihypertensives in ATC-group C02](http://en.wikipedia.org/wiki/ATC_code_C02#C02N_Combinations_of_antihypertensives_in_ATC-group_C02%23C02N_Combinations_of_antihypertensives_in_ATC-group_C02) |
| C03A | [Low-ceiling diuretics, thiazides](http://en.wikipedia.org/wiki/ATC_code_C03#C03A_Low-ceiling_diuretics.2C_thiazides%23C03A_Low-ceiling_diuretics.2C_thiazides) |
| C03B | [Low-ceiling diuretics, excluding thiazides](http://en.wikipedia.org/wiki/ATC_code_C03#C03B_Low-ceiling_diuretics.2C_excluding_thiazides%23C03B_Low-ceiling_diuretics.2C_excluding_thiazides) |
| C03C | [High-ceiling diuretics](http://en.wikipedia.org/wiki/ATC_code_C03#C03C_High-ceiling_diuretics%23C03C_High-ceiling_diuretics) |
| C03D | [Potassium-sparing agents](http://en.wikipedia.org/wiki/ATC_code_C03#C03D_Potassium-sparing_agents%23C03D_Potassium-sparing_agents) |
| C03E | [Diuretics and potassium-sparing agents in combination](http://en.wikipedia.org/wiki/ATC_code_C03#C03E_Diuretics_and_potassium-sparing_agents_in_combination%23C03E_Diuretics_and_potassium-sparing_agents_in_combination) |
| C07A | [C07A Beta blocking agents](http://en.wikipedia.org/wiki/ATC_code_C07#C07A_Beta_blocking_agents%23C07A_Beta_blocking_agents) |
| C07B | [Beta blocking agents and thiazides](http://en.wikipedia.org/wiki/ATC_code_C07#C07B_Beta_blocking_agents_and_thiazides%23C07B_Beta_blocking_agents_and_thiazides) |
| C07C | [Beta blocking agents and other diuretics](http://en.wikipedia.org/wiki/ATC_code_C07#C07C_Beta_blocking_agents_and_other_diuretics%23C07C_Beta_blocking_agents_and_other_diuretics) |
| C07D | [Beta blocking agents, thiazides and other diuretics](http://en.wikipedia.org/wiki/ATC_code_C07#C07D_Beta_blocking_agents.2C_thiazides_and_other_diuretics%23C07D_Beta_blocking_agents.2C_thiazides_and_other_diuretics) |
| C07E | [Beta blocking agents and vasodilators](http://en.wikipedia.org/wiki/ATC_code_C07#C07E_Beta_blocking_agents_and_vasodilators%23C07E_Beta_blocking_agents_and_vasodilators) |
| C07F | [Beta blocking agents and other antihypertensives](http://en.wikipedia.org/wiki/ATC_code_C07#C07F_Beta_blocking_agents_and_other_antihypertensives%23C07F_Beta_blocking_agents_and_other_antihypertensives) |
| C08C | [Selective calcium channel blockers with mainly vascular effects](http://en.wikipedia.org/wiki/ATC_code_C08#C08C_Selective_calcium_channel_blockers_with_mainly_vascular_effects%23C08C_Selective_calcium_channel_blockers_with_mainly_vascular_effects) |
| C08D | [Selective calcium channel blockers with direct cardiac effects](http://en.wikipedia.org/wiki/ATC_code_C08#C08D_Selective_calcium_channel_blockers_with_direct_cardiac_effects%23C08D_Selective_calcium_channel_blockers_with_direct_cardiac_effects) |
| C08E | [Non-selective calcium channel blockers](http://en.wikipedia.org/wiki/ATC_code_C08#C08E_Non-selective_calcium_channel_blockers%23C08E_Non-selective_calcium_channel_blockers) |
| C08G | [Calcium channel blockers and diuretics](http://en.wikipedia.org/wiki/ATC_code_C08#C08G_Calcium_channel_blockers_and_diuretics%23C08G_Calcium_channel_blockers_and_diuretics) |
| C09A | [ACE inhibitors, plain](http://en.wikipedia.org/wiki/ATC_code_C09#C09A_ACE_inhibitors.2C_plain%23C09A_ACE_inhibitors.2C_plain) |
| C09B | [ACE inhibitors, combinations](http://en.wikipedia.org/wiki/ATC_code_C09#C09B_ACE_inhibitors.2C_combinations%23C09B_ACE_inhibitors.2C_combinations) |
| C09C | [Angiotensin II antagonists, plain](http://en.wikipedia.org/wiki/ATC_code_C09#C09C_Angiotensin_II_antagonists.2C_plain%23C09C_Angiotensin_II_antagonists.2C_plain) |
| C09D | [Angiotensin II antagonists, combinations](http://en.wikipedia.org/wiki/ATC_code_C09#C09D_Angiotensin_II_antagonists.2C_combinations%23C09D_Angiotensin_II_antagonists.2C_combinations) |
| C09X | [Other agents acting on the renin-angiotensin system](http://en.wikipedia.org/wiki/ATC_code_C09#C09X_Other_agents_acting_on_the_renin-angiotensin_system%23C09X_Other_agents_acting_on_the_renin-angiotensin_system) |
| C10A | [Lipid modifying agents, plain](http://en.wikipedia.org/wiki/ATC_code_C10#C10A_Lipid_modifying_agents.2C_plain%23C10A_Lipid_modifying_agents.2C_plain) |
| C10B | [Lipid modifying agents, combinations](http://en.wikipedia.org/wiki/ATC_code_C10#C10B_Lipid_modifying_agents.2C_combinations%23C10B_Lipid_modifying_agents.2C_combinations) |
| D03A | Cicatrizants |
| D03B | Enzyms |
| D06A | Antibiotics for Topical Use |
| D06B | Chemotherapeutics for Topical Use |
| G04BE | Drugs used in [erectile dysfunction](http://en.wikipedia.org/wiki/Erectile_dysfunction) |
| N07BA | Drugs used in nicotine dependence |
